# Immunomodulatory activity of geranial, geranial acetate, gingerol, and eugenol essential oils: evidence for humoral and cell-mediated responses

**Published:** 2013

**Authors:** Seema Farhath, PP Vijaya, Manivannan Vimal

**Affiliations:** 1 *Research Scholar, Research and Development, Bharathiar University, Coimbatore, Tamilnadu, India - 641 046*; 2 *Department of Biotechnology, Mohamed Sathak College of Arts and Science, Chennai, India- 119. Sholinganallur, Chennai, Tamil Nadu, India *

**Keywords:** Geranial, Geranial acetate, Gingerol, Eugenol, Haemagglutination

## Abstract

**Objective: **The immunomodulatory effect of geranial, geranial acetate, gingerol, and eugenol essential oils were evaluated by studying humoral and cell-mediated immune responses.

**Materials and Method:** The essential oils were evaluated for immunomodulatory activity in *in vivo* studies, using rats as the animal model. The essential oils were tested for hypersensitivity and hemagglutination reactions, using sheep red blood cells (SRBC) as the antigen while sodium carboxy methyl cellulose (SCMC) served as the control in all the tests.

**Result:** Orally administrated essential oils showed a significant increase of test parameters, viz., haemagglutinating antibody titre (HAT) and delayed type hypersensitivity (DTH) response. In rats immunized with sheep RBC, essential oils enhanced the humoral antibody response to the antigen and significantly potentiated the cellular immunity by facilitating the foot pad thickness response to sheep RBC in sensitized rats with doses of 50-800 mg/ml. Haemagglutination titre of geraniol showed the highest increase of 139.3±6.38 and with 5.9±0.7 DTH, respectively. For geranial acetate, the haemagglutination titre showed a moderate increase of 87.5±5.9 and highest increase in DTH with 5.9±0.8, respectively. Using gingerol, the haemagglutination titre showed a moderate increase with 88.2±6.306 and DTH 3.5±0.5, respectively and for eugenol, the haemaggulation titre showed a moderate increase with 112.06±6.169 and DTH 4.4±0.6, respectively. These differences were statistically significant.

**Conclusion:** The essential oils were found to have a significant immunostimulant activity on both the specific and non-specific immune mechanisms.

## Introduction

There has been an increasing interest in the use of natural substances especially, essential oils, odors, and volatile products of plants secondary metabolism which have a wide application in folk medicine as well as in fragrance industries. Essential oils are complex natural mixtures of volatile secondary metabolites, isolated from plants by hydro- or steam-distillation. The main constituents of essential oils, for example, monoterpenes and sesquiterpenes, and phenylpropanoids including carbohydrates, alcohols, ethers, aldehydes, and ketones, are responsible for the fragrant and biological properties of aromatic and medicinal plants (Raphael et al., 2003 ▶). 

The immunomodulatory activity of some naturally occurring monoterpenes such as carvone, limonene, and perillic acid were also studied (Reichling et al., 1999 ▶).Various essential oils and their components possess pharmacological effects, demonstrating anti-inflammatory, antioxidant, and anti-carcinogenic properties (Golab et al., 2005 ▶). In addition to inducing resistance, antibiotics are sometimes associated with opposing effects such as hypersensitivity, immune-suppression, and allergic reactions (Ahmad et al., 1998 ▶). Therefore, there is a need to develop alternative drugs for the treatment of infectious diseases (Berahou et al., 2007 ▶).

It is important to investigate scientifically those plants which have been used in traditional medicines and play a vital role in immune system. Moreover, the resurgence of interest in natural therapies and increasing consumer demand for effective and safe natural products mean that quantitative data on plant oils and extracts are required. Various studies have been documented immunomodulatory effect of essential oils and plant extracts including ginger, sage, clove oil, and tea oil (Carrasco et al., 2009 ▶; Golab et al., 2005 ▶). 

The main advantage of essential oils is that they can be used in any food and are considered generally recognized as safe (GRAS) as long as their maximum effects are attained with the minimum change in the organoleptic properties of the food (Nelson et al., 1967 ▶). 

The current study aimed at exploring the immunomodulatory potential of the essential oils, geranial, geranial acetate, gingerol, and eugenol.

## Materials and Methods

**Essential oil compounds**

Four essential oil compounds obtained from Commercial producers of plant essential oils and aromatic substances (Sigma & Co (P) Ltd, India) were used in this study. Quality of the oils was ascertained by GC to be more than 98% pure. 

**Animals**

Male rats (10 weeks old and between 220 and 260 g in body weight) were used for acute toxicity and pharmacological studies. The animals were maintained at room temperature and fed with standard pellet diet (Lipton India Ltd.) and tap water, ad libitum. The studies were approved by the Institutional Animals Ethical Committee.

**Dose fixation**

Doses of the test formulations were calculated by extrapolating the human dose to animals, based on the body surface area ratio referring to the standard table of Paget and Barnes (1969) ▶. The test formulation was suspended in distilled water and administered orally at a volume of 0.5 ml/100 g body weight with the help of gastric catheter of suitable size sleeved on to a syringe nozzle to overnight fasted animals.

**Antigen**

Fresh blood was collected from sheep sacrificed in the local slaughter house. Sheep red blood cells (SRBCs) were washed three times in normal saline and adjusted to a concentration of 0.1 ml containing 1×10^8 ^cells for immunization and challenge.

**Humoral antibody (HA) response**

Humoral antibody (HA) response was identified using the method described by ham agglutination technique (Nelson et al 1967 ▶). Mice were divided into seven groups, each group containing six mice. Drugs were administered to various groups, i.e., Group I: control group (Sodium carboxy methyl cellulose (SCMC) 1%), Groups II–VI: test extracts I (5 dose levels 50–800 mg/kg p.o.), and Group VII: standard drug (Cyclophosphamide 50 mg/kg, p.o.). On the 7^th^ and 15^th^ day of the study, the rats from all of the groups were immunized and challenged respectively with SRBCs in normal saline (0.1 ml of suspension containing 1×10^8^ SRBC) intraperitoneally. 

Blood was withdrawn from the retro orbital plexus from all antigenically sensitized and challenged rats on day 14 and centrifuged to get serum. The blood samples were centrifuged and serum was obtained. Antibody levels were determined by the haemagglutination technique. Briefly, equal volumes of individual serum samples of each group were pooled. To the serial of two-fold dilutions, pooled serum samples made in 25 µL of 1% suspension of SRBCs in saline is used. After mixing, the plates were incubated at 37 ^o^C for 1 h and examined for haemagglutination under microscope. The reciprocal of the highest dilution of the test serum giving agglutination was taken as the antibody titre.

**Delayed type hypersensitivity (DTH)**

Hypersensitivity reaction to SRBC was induced in rats, following the prescribed method (Doherty 1981 ▶). Delayed type hypersensitivity was assessed using mice. On day 7, the thickness of the right hind foot pad was measured using vernier caliper. The mice were then challenged by injection of 1×10^7-8 ^SRBCs in right hind foot pad. Foot thickness was measured again 24 h after this challenge. The difference between the pre- and post-challenge foot thickness expressed in mm was taken as a measure of DTH. The extract was administered orally on day 0 and continued till day 7 of the challenge. Cyclophosphamide was administered on days 4 to 6.

Effect of essential oil and cyclophosphamide on HA titre and DTH response was observed using SRBCs as an antigen in mice – 7 days pre-treatment. Mice were divided into six groups, each group containing six mice. The Group I was the control (Sodium carboxy methyl cellulose 1%), Groups II-VI received essential oil compounds I (5 dose levels 50-800 mg/kg p.o.). Pretreatment time of 15 days was treated to the mice. Schedule for drug administration was 7 days prior to immunization (days - 6, - 5, -4, -3, -2, -1, and 0) and 7 days after immunization (days +1, +2, +3, +4, +5, +6, and +7). The extent of DTH response in rats was determined by measuring the footpad thickness after 7^th^ and 15^th^ day of challenge. 

## Results

**Pharmacological investigations haemagglutination reaction**

The antigen antibody reaction results in agglutination. The relative strength of an antibody titre is defined as the reciprocal of the highest dilution which is still capable of causing visible agglutination. The antibody titre is useful to measure the changes in the amount of the antibody in the course of an immune response.Administration of the essential oils (50, 100, 200, 400, and 800 mg/kg) for seven days produced a dose related increase in the antibody titre in rats (Table 1).

Administration of the essential oils (50, 100, 200, 400, and 800 mg/kg) for 15 days produced a dose-related increase in the antibody titre in rats (Table 2).

**Table 1:** Effect of essential oil in HA titre using SRBCs as an antigen in mice, 7-days treatment

**Groups/dose ** **mg/kg**		**Geraniol**	**Geranial acetate**	**Gingerol**	**Eugenol**
**I ** **–** ** Control**		7.9±0.93	7.9±0.138	7.9±0.146	7.9±0.111
**II ** **–** ** 50 **		8.8±0.89	7.9±0.138	8.1±0.161	15.9±0.161
**III ** **–** ** 100**		14.7±0.69	15.8±0.712	13.7±0.591	32.2±1.525
**IV ** **–** ** 200**		16±0.479	51.5±3.306	31.3±1.941	33.0±1.571
**V- 400**		41.2±1.68	65.5±4.322	53.7±3.660	88.6±4.807
**VI ** **–** ** 800**		139.3±6.38	87.5±5.922	88.2±6.306	112.0±6.169
**ANOVA**	** F**	**4.673**	**3.198**	**3.782**	**3.548**
	df	5,20	5,20	5,20	5,20
	P	<0.05	<0.05	<0.05	<0.05

Values are mean±SEM, n=6 in each group, *p<0.05 when compared with respective control group (Dunnett’s test).

**Table 2:** Effect of essential oil in HA titre using SRBCs as an antigen in mice, 15-days treatment

**Groups/dose** **mg/kg**		**Geraniol**	**Geranial acetate**	**Gingerol**	**Eugenol**
**I ** **–** ** Control**		16.5±0.530	26.2±1.212	6.6±0.0616	16.3±0.353
**II ** **–** ** 50 **		52.1±2.328	52.6±2.796	16.1±1.037	52.4±1.590
**III ** **–** ** 100**		65.8±3.019	72.4±3.984	17.2±1.150	85.5±2.723
**IV ** **–** ** 200**		97.5±4.620	99.7±5.622	24.3±1.878	120.6±3.926
**V- 400**		108.1±5.155	114.4±6.503	53.5±4.876	152.7±5.025
**VI ** **–** ** 800**		155.5±7.548	136.0±7.799	66.9±6.251	214.8±7.153
**ANOVA**	** F**	** 4.673**	**2.854**	** 1.205 **	**2.950**
	df	5,20	5,20	5,20	5,20
	P	<0.05	<0.05	<0.05	<0.05

Values are mean±SEM, n=6 in each group, *p<0.05 when compared with respective control group (Dunnett’s test).

Daily administration of geraniol oil for 7 consecutive days produced a significant (p<0.05) increase in humoral antibody titre at (50-800 µg/µl), but very meager increase was observed at the higher dose with 155.5±7.548 at 15^th^ day of treatment. Geranial acetate (50-800 µg/µl) produced a dose-related increase (p<0.05) in antibody titre compared to the control rats and a significant difference was observed between the two doses with 87.5±5.922 and 136±7.799, respectively when compared with each other. Gingerol (50-800 µg/µl) showed an increase in humoral titre with 88.2±6.306 but comparatively smaller than other essential oils at the higher dose of 800 µg/µl and there was no significant increase in 15^th^ day of treatment. Eugenol showed a significant increase in both in 7^th^ and 15^th^ day of treatment with 1126.169 and 214.8±7.153, respectively.

**Hypersensitivity reaction**

DTH plays a major role in understanding processes such as graft rejection, tumour immunity, and immunity to many intracellular infectious microorganisms such as tuberculosis (Elgert et al., 1996 ▶). Oral administration of the essential oils (50, 100, 200, 400, and 800 mg/kg) for 7 days produced a dose-related increase in early (7^th^ day) and delayed (15^th^ day) hypersensitivity reaction in rats. The 15^th^ day was found to be of higher magnitude than the 5^th^ day. (Tables 3 and 4). These results indicate that the Geraniol acetate has a greater effect on the delayed hypersensitivity reaction with a p-value 2.086 with a moderate effect on other essential oils. The thickness of the hind paw challenged with sheep RBC in the geraniol acetate treated group was almost 54% more (5.6±0.7) than that in the gingerol group.

**Table 3:** Effect of essential oil in DTH response using SRBCs as an antigen in mice, 7-days treatment

**Groups/dose** **mg/kg**		**DTH response, mean paw edema in mm**			
		**Geraniol**	**Geranial acetate**	**Gingerol**	**Eugenol**
**I ** **–** ** Control**		0.2±0.03	0.2±0.02	0.2±0.118	0.25±0.02
**II ** **–** ** 50 **		3.9±0.3	2.8±0.23	3.2±0.3	2.2±0.2
**III ** **–** ** 100**		4.3±0.4	2.9±0.3	3.0±0.4	2.4±0.2
**IV ** **–** ** 200**		4.4±0.4	5.1±0.7	3.5±0.5	3.6±0.5
**V- 400**		5.1±0.6	5.2±0.8	3.3±0.4	3.8±0.5
**VI ** **–** ** 800**		5.6±0.7	5.9±0.8	3.5±0.5	4.4±0.6
**ANOVA**	**F**	**1.518**	**2.086**	**1.230 **	** 2.011**
	df	5,20	5,20	5,20	5,20
	P	<0.05	<0.05	<0.05	<0.05

Values are mean±SEM, n=6 in each group, *p<0.05 when compared with respective control group (Dunnett’s test).

**Table 4:** Effect of essential oil in DTH response using SRBCs as an antigen in mice, 15-days treatment

**Groups/dose** **mg/kg**		**DTH response-mean paw edema in mm**			
		**Geraniol**	**Geranial acetate**	**Gingerol**	**Eugenol**
**I ** **–** ** Control**		0.2±0.03	0.2±0.03	0.2±0.118	0.25±0.02
**II ** **–** ** 50 **		5.0±0.3	4.0±0.2	3.0±0.4	4.9±0.3
**III ** **–** ** 100**		5.0±0.5	4.1±0.4	5.9±0.9	5.0±0.5
**IV ** **–** ** 200**		5.2±0.6	4.3±0.6	5.9±0.9	5.2±0.5
**V- 400**		5.9±0.8	5.6±0.8	6.2±1.01	5.3±0.6
**VI ** **–** ** 800**		6.2±0.9	5.7±0.8	6.3±1.0212	5.9±0.8
**ANOVA**	** F**	**1.361**	**1.648**	**1.560 **	**1.286**
	df	5,20	5,20	5,20	5,20
	P	<0.05	<0.05	<0.05	<0.05

Values are mean±SEM, n=6 in each group, *p<0.05 when compared with respective control group (Dunnett’s test).

## Discussion

The present study demonstrates the immunostimulant potential of the essential oils. As the essential oils showed promising immunostimulant activity in the *in vitro* test, it was time for *in vivo* animal studies. The results of *in vivo* animal studies showed an increase in the early and delayed hypersensitivity reaction to SRBC at doses of 100, 200, 400, and 800 mg/kg. This indicated the stimulatory effect of essential oils on chemotaxis-dependent leucocytes migration. In the early hypersensitivity reaction, the antigen antibody forms immune complexes, which are known to induce local inflammation with increased vascular permeability and edema.

Antibody molecules which are secreted by plasma cells mediate the humoral immune response. The essential oils showed an increase in the haemagglutination titre at doses of 100, 200, 400, and 800 mg/kg in animal studies. This augmentation of the humoral response to SRBC indicated an enhanced responsiveness of the macrophages and T and B lymphocyte subsets involved in antibody synthesis (Benacerraf et al., 1978 ▶).

Result of our study revealed that, the foot pat thickness of essential oils (p<0.05) significantly enhanced the production of circulating antibody titre in response to sheep red blood cells (SRBC) and phagocytic functions of mononuclear macrophages and non-specific immunity. Result were also supported by haemaggulutination tests data. Hence, the present investigation reveals that, essential oils possess immunostimulant properties. The essential oils were found to have a significant immunostimulant activity on both specific and non-specific immune mechanisms. Geraniol and geraniol acetate were found to have a promising immunomodulatory effect than gingerol and eugenol. In conclusion, this *in vitro* study revealed the capacity of all of the studied essential oils to enhance the proliferation of lymphocytes.
